# Multi-year crop rotation and quicklime application promote stable peanut yield and high nutrient-use efficiency by regulating soil nutrient availability and bacterial/fungal community

**DOI:** 10.3389/fmicb.2024.1367184

**Published:** 2024-05-17

**Authors:** Liyu Yang, Caibin Wang, Xinhua He, Haiyan Liang, Qi Wu, Xuewu Sun, Miao Liu, Pu Shen

**Affiliations:** ^1^Shandong Peanut Research Institute/Key Laboratory of Peanut Biology, Genetic & Breeding, Ministry of Agriculture and Rural Affairs, Shandong Academy of Agricultural Sciences, Qingdao, Shandong, China; ^2^College of Resources and Environment, Southwest University, Chongqing, China

**Keywords:** peanut, crop rotation pattern, continuous cropping obstacle, bacterial/fungal community, quicklime

## Abstract

Diversifying cultivation management, including different crop rotation patterns and soil amendment, are effective strategies for alleviating the obstacles of continuous cropping in peanut (*Arachis hypogaea* L.). However, the peanut yield enhancement effect and temporal changes in soil chemical properties and microbial activities in response to differential multi-year crop rotation patterns and soil amendment remain unclear. In the present study, a multi-year localization experiment with the consecutive application of five different cultivation managements (including rotation with different crops under the presence or absence of external quicklime as soil amendment) was conducted to investigate the dynamic changes in peanut nutrient uptake and yield status, soil chemical property, microbial community composition and function. Peanut continuous cropping led to a reduction in peanut yield, while green manure-peanut rotation and wheat-maize-peanut rotation increased peanut yield by 40.59 and 81.95%, respectively. A combination of quicklime application increased yield by a further 28.76 and 24.34%. Alterations in cultivation management also strongly affected the soil pH, nutrient content, and composition and function of the microbial community. The fungal community was more sensitive than the bacterial community to cultivation pattern shift. Variation in bacterial community was mainly attributed to soil organic carbon, pH and calcium content, while variation in fungal community was more closely related to soil phosphorus content. Wheat-maize-peanut rotation combined with quicklime application effectively modifies the soil acidification environment, improves the soil fertility, reshapes the composition of beneficial and harmful microbial communities, thereby improving soil health, promoting peanut development, and alleviating peanut continuous cropping obstacles. We concluded that wheat-maize-peanut rotation in combination with quicklime application was the effective practice to improve the soil fertility and change the composition of potentially beneficial and pathogenic microbial communities in the soil, which is strongly beneficial for building a healthy soil micro-ecology, promoting the growth and development of peanut, and reducing the harm caused by continuous cropping obstacles to peanut.

## Introduction

Peanut is one of the most widely cultivated economic crops and an important source of edible oil in the world (Bertioli et al., 2016). The global demand for peanut has continued to grow over the past decade. Recently, peanut continuous cropping has become common due to the growing land crisis for peanut production. However, continuous cropping leads to peanut growth and development abnormalities, severe diseases, significant yield reductions, posing a risk to the supply of edible oils (Chen et al., 2014; Li et al., 2018, 2022; Yu et al., 2024). As a result, selecting suitable cropping method to eliminate the adverse effects of continuous cropping obstacles is a challenge for the peanut plantation and industry.

Recent evidence have indicated that the overall alteration of the soil microcosm system was the leading cause of continuous cropping obstacles (Liu et al., 2020, 2021; Xiao et al., 2024). This is represented by the imbalance of soil nutrients and soil microbiota, self-toxic effect of chemo-sensitive substances, and alteration of soil pH (Shen et al., 2019). After growing the same crop continuously for a long time, specific nutrient is selectively absorbed by and removed from crops, resulting in nutrient imbalance in soil (Pervaiz et al., 2020). Besides nutrient imbalance, continuous cropping is often accompanied by soil acidification. Numerous studies have shown that plant roots secreted organic acids during growth, and long-term cultivation of the same crop could lead to the accumulation of specific organic acids in soil (Tan et al., 2017; Bai et al., 2019). Nitrogen (N) is an important nutrient for the development of most crops, and the selective uptake of NH_4_^+^ by some crops including rice, maize, and sorghum results in a persistent deficit of NH_4_^+^ in soil, which produce saline acidity and further aggravate the acidification in continuous cropping soil (Fageria and Baligar, 2005). In addition, some secondary metabolites, such as phenols and terpenoids, are secreted by root systems during crop development and have very strong chemosensory activity. With the increase of cropping years, these chemo-sensitive substances will accumulate in soil and become self-toxic to crop (including peanut), making the crop less resistant, decreasing the yield and further aggravating the continuous cropping obstacles (Li et al., 2014a).

Soil microorganisms are directly involved in decomposing of soil organic matter, nutrient transformation and soil-borne diseases (Fierer et al., 2020). In recent years, increased studies showed that soil microflora dysbiosis was a fundamental cause of continuous cropping obstacles. For instance, the diversity, function and co-occurrence networks of soil fungi and bacteria were significantly affected by continuous cropping with interannual variations (Liu et al., 2020, 2021). The dominant fungi from the continuous cropping soil showed significantly inhibitory effects on the growth and development of crops, further suggesting that the altered microorganisms caused by continuous cropping was the fundamental cause of constant cropping obstacles (Liu et al., 2021). Studies also showed that a strong correlation exists between continuous cropping obstacles in peanut plantation with changes in soil microbial communities and compositions (Li et al., 2018, 2022). With continuously increasing cropping years, the number of pathogenic fungi in soil and inter-rhizosphere also increased, while the number of bacteria and actinomycetes decreased significantly. The abundance and/or diversity of plant beneficial bacteria including *Alteromonadales*, *Burkholderiales*, *Flavobacteriales*, *Pseudomonadales*, *Rhizobiales*, and *Rhodospirillales*, have a decreased propensity with continuous cropping (Chen et al., 2014). At the same time, fungi in soil trended to increase pathogenic fungi and simplify of beneficial fungal communities (Chen et al., 2012; Li et al., 2014b). Li et al. (2018) treated peanut seedling with bacterial suspensions extracted from continuous-cropping soil and found that such bacterial suspensions markedly suppressed peanut growth. Therefore, an imbalance in the soil microbial community structure can be a leading cause of continuous cropping obstacles for peanut.

The formation of continuous cropping obstacles is related to the deterioration of soil chemical properties and microbial community structure disorders. Therefore, applying rational cultivation patterns to improve soil chemical properties and change microbial community structure becomes an effective approach to eliminate continuous cropping obstacles in agricultural production (Kagawa et al., 2022; Wang et al., 2022). It has shown in studies that changes in cultivation managements (including the reasonable application of crop rotation and soil amendments) can effectively reduce the harmful effects of continuous cropping obstacles (Aparicio and Costa, 2007; Huang et al., 2015; Zhang et al., 2022a). Reasonable crop rotation is an effective agronomic management strategy to reduce continuous cropping obstacles. The multiple crops introduced by crop rotation can balance soil nutrients and improve soil fertility due to different nutrient ecological niches (Passaris et al., 2021). Moreover, by continuously changing hosts, it can break the specialized parasitic pathogens and oligophagous pests, and reduce the pest and disease hazards caused by continuous cropping (Carrière et al., 2020). The different inter-root environments brought by different crops are conducive to the construction of a healthy soil microbial community, further reducing the damage caused by continuous cropping (He et al., 2019). Differences in the crop species used for crop rotation make different crop rotation patterns have different mitigating effects on the cropping obstacles (Venter et al., 2016; Sumner, 2018; Massigoge et al., 2024). Green manure rotation and wheat-maize rotation are the two main crop rotation patterns in peanut cultivation process, however, the literature on effect of different crop rotation patterns on the alleviation of continuous cropping obstacles in peanut and the underlying mechanism of its regulation are lacking is scarce. Quicklime is an important soil amendment. Applying quicklime by increasing external calcium to soil is a common method of soil disinfection and plays an essential role in increasing soil pH of acid soil caused by continuous cropping, and reconstructing the composition structure of microorganisms, mitigating the continuous cropping obstacles to crop production (You et al., 2015; Lu et al., 2021). Exploring effective agricultural management measures is a meaningful way to alleviate and eliminate continuous cropping obstacles. Recently, although studies have recently focused on the effects of various cultivation managements on peanut continuous cropping obstacles, there is still a paucity of evidence regarding how the multi-year cultivation management affects peanut growth, soil chemical properties, and microbial community structure in peanut continuous cropping system.

To explore the characteristics of peanut continuous cropping obstacles and the intrinsic mechanisms in the application of different cultivation managements (including rotation with different crops under the presence or absence of external quicklime) for consecutive years to alleviate peanut continuous cropping obstacles, we investigate the effects of different cultivation managements on peanut yield, soil chemical properties and microbial community composition and function in peanut cropping soil at different cropping year. The effects of different long-term cultivation management on peanut growth performance and soil nutrient availability were assessed by measuring the yield and nutrient of peanut and determining properties of soil organic matter, available nitrogen, phosphorus, potassium and calcium nutrients. Bacterial and fungal communities in the soil were determined using Illumina MiSeq platform. We hypothesize that different cultivation management and cropping year could alter soil chemistry and shape microbial community structure, thereby offering a beneficial environment for peanut growth, alleviating peanut continuous cropping obstacles, and thus increasing the yield and nutrient content of peanut. This study increases our understanding of the mechanism of different cultivation management that change soil chemical properties and drive alternations in the composition and function of the microbial community to mitigate continuous cropping obstacles for peanut. The results of this study will provide a theoretical and practical basis for high-quality cultivation and sustainable peanut production.

## Materials and methods

### Study site description and experimental design

This multi-year localization experiment was conducted at a typically acidic brown soil (*Haplic Luvisols*, the FAO soil classification system) in a major peanut production area, Laixi County, Shandong (E120^o^29’, N36^o^ 48′, 227 m above the sea level), China. At the experimental sites peanut has never been planted in this brown soil that is developed from weathered acid parent rocks. The average annual temperature was 11.5°C, and the average annual rainfall was 635.8 mm.

In a randomly complete block design with three replicates for each treatment, five continuously experimental treatments were examined: (1) T1, annual summer peanut and winter fallow; (2) T2, annual summer peanut and winter ryegrass without quicklime application; (3) T3, annual summer peanut and winter ryegrass plus quicklime application; (4) T4, summer peanut rotated with maize and winter wheat; (5) T5, summer peanut rotated with maize and winter wheat plus quicklime application. Nitrogen, phosphorus (P), and potassium (K) fertilizers were based on the local fertilization practice. With 600 kg CaO (quicklime)/hm^2^, a total of 750 kg/hm^2^ ternary compound fertilizer (15%N: 15% P_2_O_5_: 15% K_2_O) were applied containing 112.5 kg N/hm^2^, 112.5 kg P_2_O_5_/hm^2^, 112.5 kg K_2_O/hm^2^.

### Soil and plant sampling and analyses

A common local peanut variety of Huayu 33 was seeded in early May and harvested in mid-September for selected experimental years. There was one peanut seed in each plant hole with a planting distance of 15 cm. Sowing rate 180,000 plants/m^2^. Soil at year1 (Base), year 3 (T1-3Y, T2-3Y, T3-3Y, T4-3Y, T5-3Y) and 5 (T1-5Y, T2-5Y, T3-5Y, T4-5Y, T5-5Y) were collected. The basic soil chemical properties mainly included soil pH, soil organic matter (SOC), soil available nitrogen (AN), available phosphorus (AP), available potassium (AK) and calcium (Ca) nutrients. After peanut harvest, soils at 0–20 cm depth were collected with a 10 cm auger. Soil samples were air-dried and passed through a 2 mm sieve and further ground to pass a 0.25 mm sieve to determine soil properties. Soil pH was measured using a pH meter (Orion 2 Star, Thermo Fisher Scientific, United States). Organic C was determined using a potassium dichromate volumetric method (Walkley, 1935). Alkali hydrolyzed or available N was determined using 1 mol/L NaOH alkali hydrolysis, 2% boric acid absorption, and 0.01 mol/L HCl titration (Mulvaney and Khan, 2001). Available P was extracted with 0.5 mol/L NaHCO_3_ and determined by UV–vis spectrophotometry (Olsen and Sommers, 1982). Available K was extracted with 1 mol/L ammonium acetate and determined by flame photometry (Lu et al., 2024). Soil Ca^2+^ concentration was determined by inductively coupled plasma atomic emission spectrometry (Agilent ICP 720-OES, Varian Inc., Darmstadt, Germany) (Dahlquist and Knoll, 1978). The initial properties of soil were as follows: pH 5.68, SOC 1.1 g/kg, AN 61.05 mg/kg, AP37.10 mg/kg, AK 114.4 mg/kg, Ca 4.42 g/kg. Concentrations of kernel N, P, K, and Ca were determined by an inductively coupled plasma atomic emission spectrometry (Agilent ICP 720-OES, Varian Inc., Darmstadt, Germany).

### DNA extraction, amplification and sequencing

Total genomic DNA of 33 soil samples was extracted separately using the Fast DNA® Spin Kit for Soil (MP Biomedicals, United States) according to the manufacturer’s instructions. The concentration and purity of DNA were then examined with 1% agarose gels. The V3-V4 region of the bacterial 16S rRNA gene was amplified using primers 515F (5′-GTGCCAGCMGCCGCGG-3′) and 806R (5′-GGACTACH VGGGTWTCTAAT-3′). The ITS1 region of the fungal gene was amplified using the primers ITS1F (5′-CTTGGTCATTTAGAGG AAGTAA-3′) and ITS2R (5′-GCTGCGTTCTTCATCGATGC-3′). All PCR reactions were conducted using a TransGen Kit (TransGen AP221-02: TransStart Fastpfu DNA Polymerase, TransGen Biotech, Beijing, China) with 20 μL reaction system, including 4 μL FastPluBuffer, 0.4 μL FastPfu Polymerase, 2 μL dNTPs, 0.8 μL of each forward and reverse primers, 0.2 μL Bovine Serum Albumin and 10 ng template DNA. DNA was amplified using ABI GeneAmp® 9,700 (Life Technologies, Foster City, CA) under the following conditions: 95°C for 5 min, followed by 95°C for 3 min, 30 cycle of 95°C for 30 s, 55°C for 30 s, 72°C for 45 s and elongation at 72°C for 10 min. After purifying, all PCR products were used to construct sequencing libraries by using the TruSeq™ DNA Sample Prep Kit (Illumina). Ultimately, the constructed libraries were sequenced on an Illumina HiSeq2500 platform at Majorbio Bio-Pharm Technology (Shanghai, China).

### Calculation and statistical analyses

After sequencing, the generated raw data were first spliced based on overlap relationships using the fast-length adjustment of short reads (FLASH) software (version 1.2.11). Using a Uparse (version 7.0.1090) software, non-repetitive sequences with >97% similarity (excluding single sequences) were categorized into operational taxonomic units (OTUs). The Ribosomal Database Project (RDP) classifier was used to analyze the taxonomy of OTU representative sequences to obtain the taxonomic information of species corresponding to each OTU. The bacteria were identified using the SILVA database,^1^ and fungi were identified using the Unite database.^2^ Alpha diversity (Shannon diversity index) (Shannon, 1948) was used to reflect the abundance and diversity of the microbial community using mothur software (version v.1.30.2). Principal coordinates analysis (PCoA) plots were used to evaluate the effects of different treatments on soil microbial composition based on the Bray-Curtis distances via “vegan” (version 2.6–4) from R package (version 3.3.1). The Mantel test analysis, redundancy analysis (RDA) and Pearson correlation heatmap were adopted to reveal the relationship between soil environmental variables and bacterial and fungal community composition. The Pearson’ s correlation coefficients were also used to investigate the correlation between environment variables environmental factors. These analyses were performed by “vegan” package and visualized by “ggcor” package (version v.0.9.8) under R environment. OTUs of bacteria were mapped to the FAPROTAX (Functional Annotation of Prokaryotic Taxa) database^3^ to predict potential metabolic functions of the detected soil bacteria. The *p*-values generated from the Welch’s t-test were used to measure the functional difference between the two different groups. Functions of identified fungi were classified via FUNGuild database^4^ (Nguyen et al., 2016). Co-occurrence network analysis was performed to detect the connections within bacterial-bacterial, bacterial-fungal and fungal-fungal taxa. The top 100 bacteria and fungi in relative abundance in each of the different libraries were selected for Person’s correlation analysis. A correction represents a strong (*r* > 0.8) and significant correlation (*p* < 0.01). The generated networks were visualized with the Gephi software (version v.0.9.6) (Bastian et al., 2009). Differences in soil properties, plant nutrients, and yield among the five treatments were subjected to analyses of variance (ANOVA) using SAS 9.4 (SAS, Inc., Cary, NC). The least significant difference (*t*-test) test was used to separate the significant differences between treatments at a 0.05 probability level.

## Results

### Effects of different cultivation managements on the pod yield of peanut

**Figure 1:**
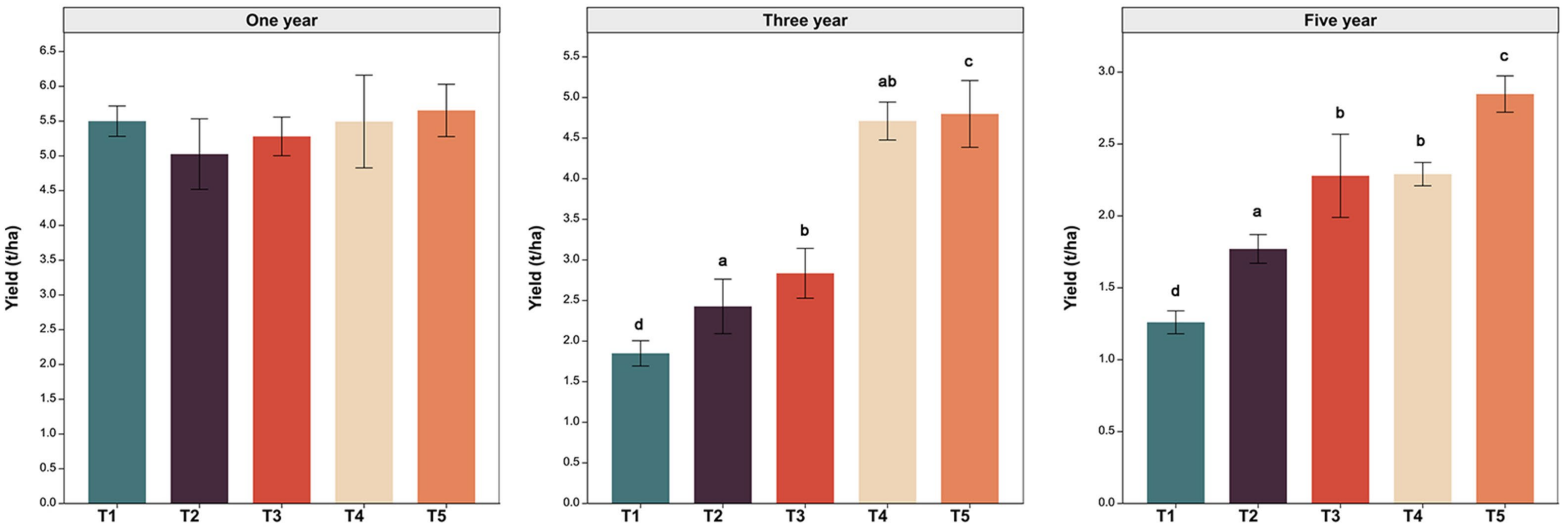
Peanut yield in year 1, 3 and 5 under five different cultivation managements. T1: No treatment; T2: Green manure rotation; T3: Green manure rotation+Quicklime; T4: Wheat-maize rotation; T5: Wheat-maize rotation +Quicklime. Different superscript letters indicate significant differences among treatments (t-test, *p* < 0.05).

The formation of peanut yield was impacted by cultivation management measures (Figure 1). In the first year of cultivation, there was no significant difference in peanut yield between treatments. In the third year of cultivation, peanut yields under the T2 (green manure rotation) and T4 treatment (wheat-maize rotation) were 1.31 and 2.55 times higher than that in T1 treatment (continuous cropping). In the fifth year of cultivation, T2 and T4 increase peanut yield by 40.59 and 81.95% compared to T1. These results indicated that crop rotation measure could significantly increase peanut yield compared to continuous cropping measures and wheat-maize rotation measure have more pronounced enhancement effect on peanut yield than green manure rotation measures. In addition, compared to treatment without quicklime (T2 and T4), peanut yield was significantly (*p* < 0.05) increased in the treatment with quicklime application (T3 and T5). At the fifth cropping year, compared to T2 and T4, T3 and T5 increase peanut yield by 28.76 and 24.34%. These results indicated that quicklime application could improve the effect of crop rotation measures on peanut yield enhancement.

### Effects of different cultivation managements on nutrient elements in peanut kernels

**Figure 2:**
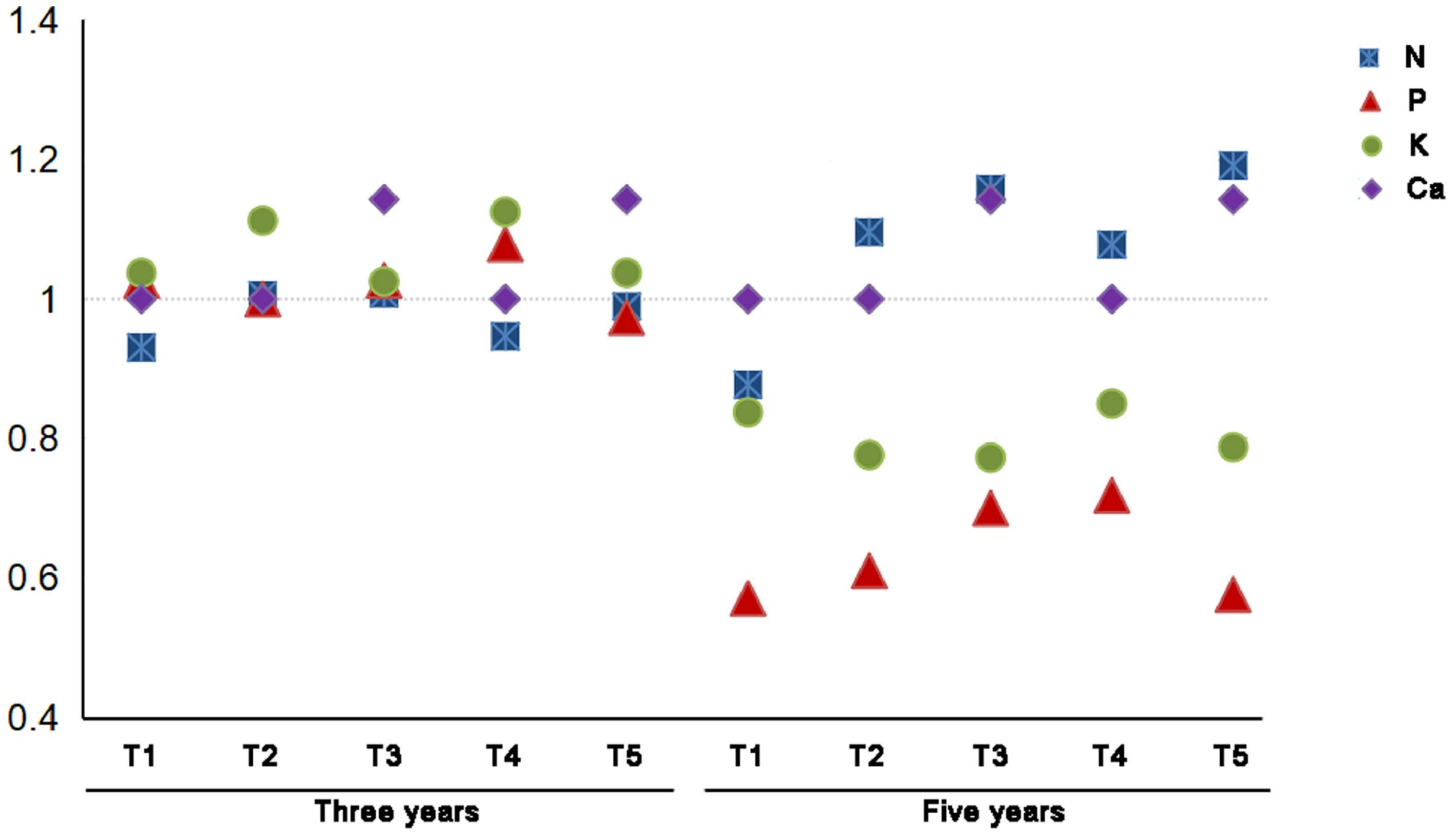
Difference of nutrient elements in peanut kernels under different cultivation managements at different cropping years. The change of ratio was relative to Base treatments.

The absorption of nutrient elements in peanut kernels was affected by continuous cropping. Different cultivation managements exhibited various effects on different nutrient elements in peanut kernels (Figure 2). Nitrogen uptake by kernel decreases with increasing years of continuous crop. Application of green manure (T2) and crop rotation (T4) could effectively improve the uptake of N by the kernels. Compared with T1-3Y, N absorption in peanut kernels increased by 8.0 and 1.7% in T3-3Y and T5-3Y. Compared with T1-5Y, N absorption increased by 25.0 and 22.9% in T3-5Y and T5-5Y, respectively. The Ca content of peanut kernels was basically unaffected by the continuous cropping years or crop rotation patterns, but was significantly increased by quicklime application.

### Effects of different cultivation managements on the soil properties

**Figure 3:**
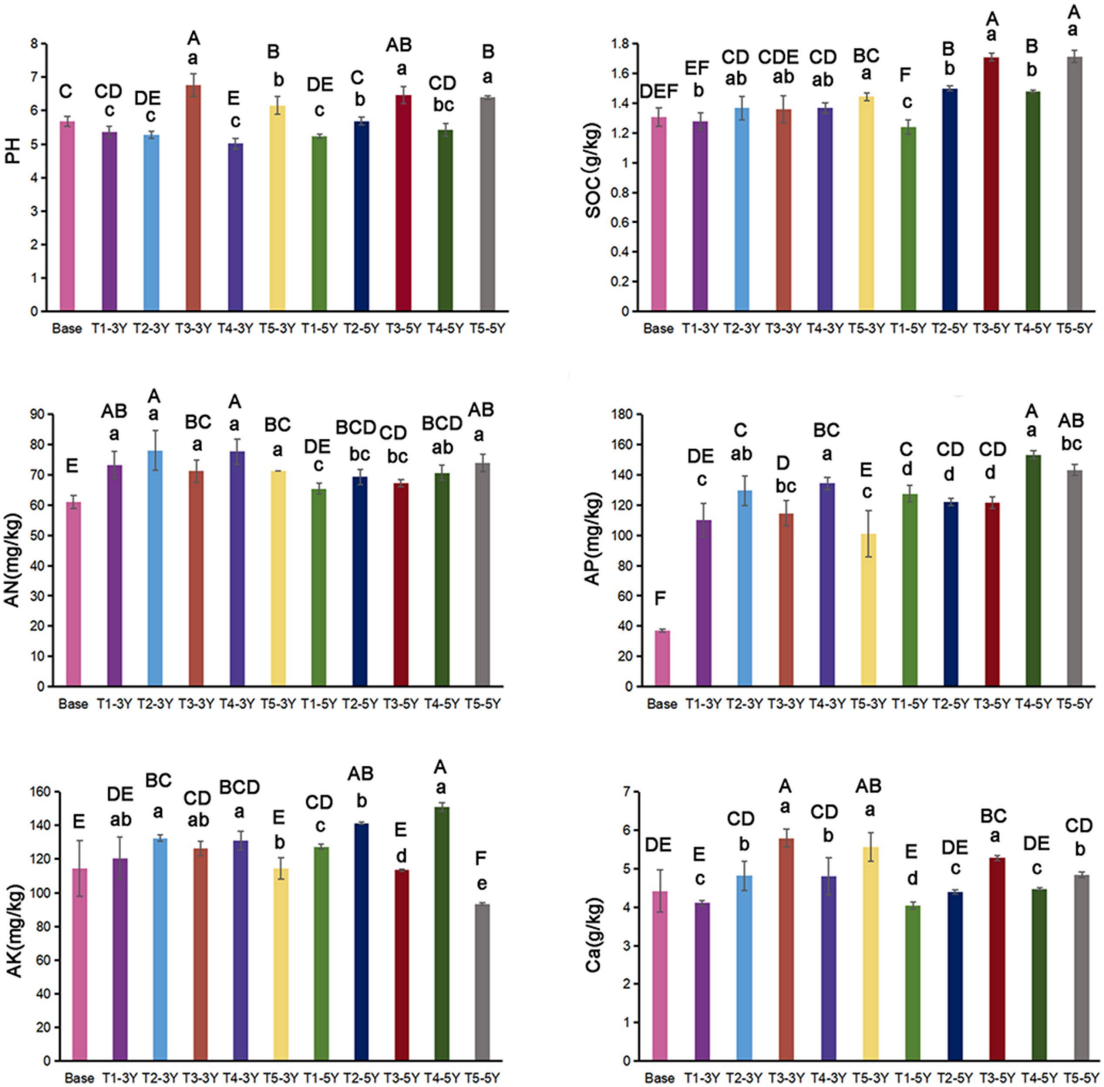
Soil properties under different cultivation managements at different cropping years. Lowercase letters indicate differences between cultivation managements in the same year, and capital letters indicate differences between all treatments.

As seen in Figure 3, soil pH exhibited a decreasing trend with increasing years of continuous cropping, by 0.31 and 0.45 units in T1-3Y and T1-5Y, compared to Base. Concentrations of SOC increased with the introduction of other crops. The highest value of AN was observed in T2-3Y. Compared with T1, soil AP and AK was elevated under T4, but decreased under a further quicklime application (T5). Soil Ca did not show significantly interannual differences. At the same cropping year, soil Ca was significantly increased by T2 (green manure rotation) and T4 (wheat-maize rotation) treatments compared to T1, and was obviously further increased with the application of quicklime.

### Differences in microbial composition under different continuous cropping years and cultivation managements

After sequencing the 16 S rRNA gene and fungal rRNA internal transcribed spacer (ITS) region, a total of 1,282,970 and 2,199,255 sequences were obtained from 11 soil samples, respectively. Soil bacterial abundance increased with the peanut cropping years, and was significantly higher at the year 5, compared to year 1 and 3 (Supplementary Table S1), as well as higher under T2-T5 than under T1. Soil fungal abundance was less affected by the peanut cultivation years. Consistent with the trend in bacterial abundance, soil fungal abundance was also higher under T2-T5 than under T1. Additionally, bacterial and fungal abundances were lower in treatments with quicklime (T3 and T5) than in those without quicklime application (T2 and T4) at the same cropping year. This result may be related to the germicidal effect of quicklime.

**Figure 4:**
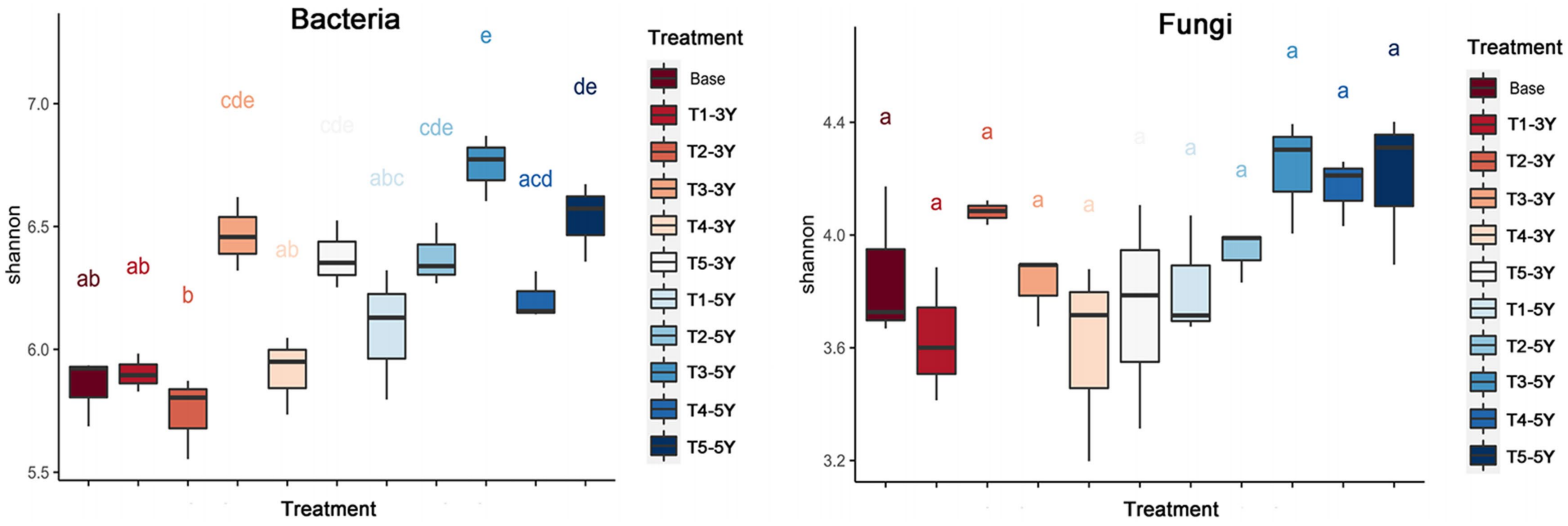
Alpha diversity of soil bacteria and fungi in each sample.

There existed differences in the α-diversity of soil bacteria and fungi under different cropping years and cultivation practice treatment conditions (Figure 4). For bacteria, by comparing the Shannon index, the diversity index of soil bacteria increased with planting years. The bacterial diversity index was significantly higher in soil samples with quicklime application than in other soil samples under the same planting year conditions, indicating that quicklime is an essential driver for increasing bacterial diversity. For fungi, the Shannon index did not differ significantly between planting years and cultivation practices. However, the Shannon index of fungi in soil with applied cultivation practices (T2-T5) was greater than that of fungi in continuous crop soils (T1) in the same cultivation years. This result indicates that continuous crop cultivation measures can decrease fungal diversity.

### Changes in microbial community composition under different continuous cropping years and cultivation managements

**Figure 5:**
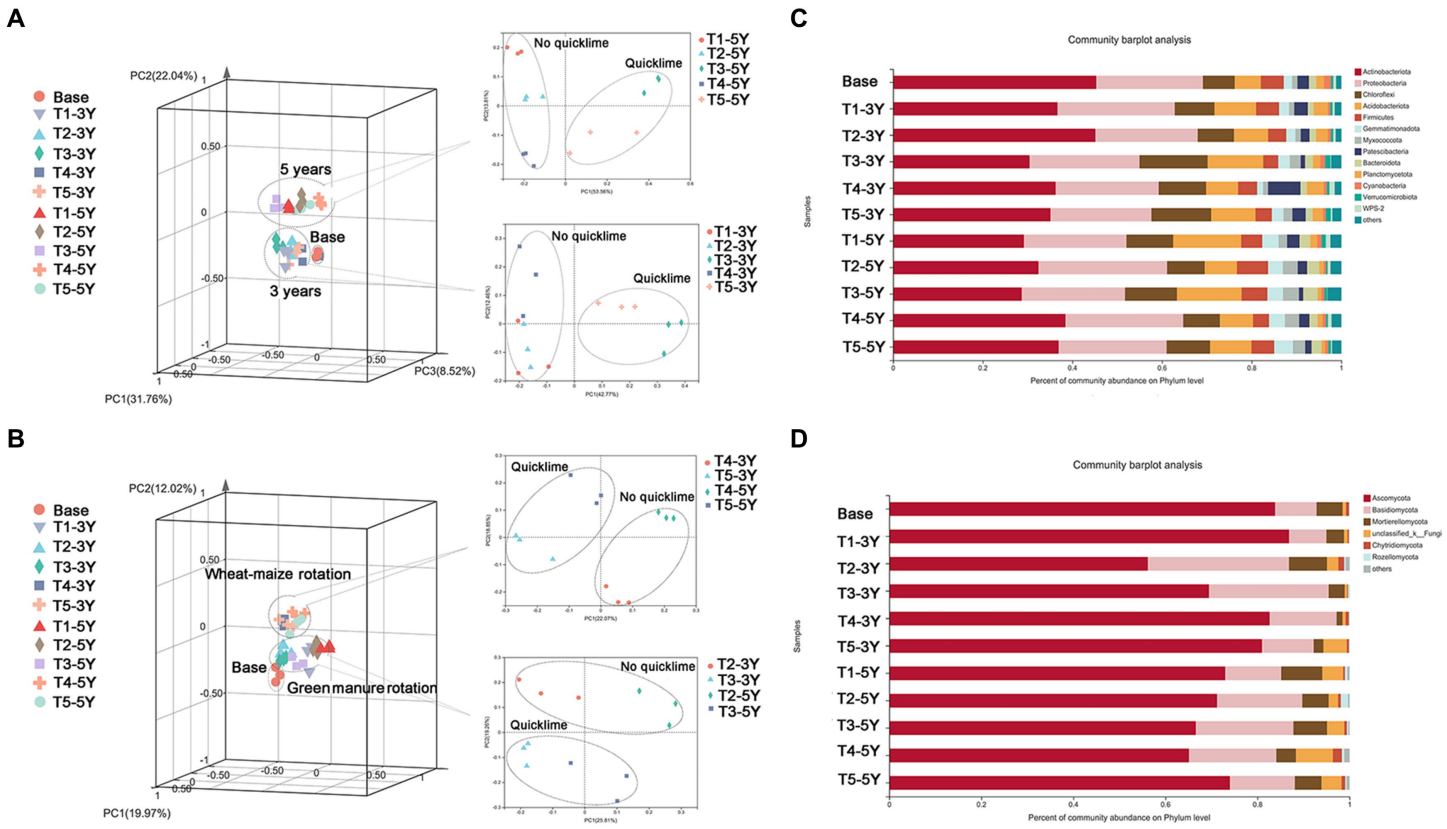
The effects of different cultivation practices on soil microbial composition. Principal coordinates analysis (PCoA) plots showing the effects of different cultivation measures on soil bacterial **(A)** and fungal **(B)** composition under different years of continuous peanut cropping based on Bray-Curtis distances. Relative abundance of bacterial **(C)** and fungal **(D)** phyla communities in each sample.

Soil bacterial and fungal communities were altered by different cultivation years and practices. In the present study, a principal coordinate analysis (PCoA) analysis was performed to measure the effects of different years and cultivation practices on the bacterial fungi in the soil. As shown in Figure 5A, the year of cultivation was the most influential factor in the structure of the soil bacterial community. Regardless of cultivation practices, soil libraries with the same cultivation years were grouped into the same cluster. In the same cultivation year, the bacterial community in the soil treated with and without quicklime was clustered into two different clusters. This indicated that the overall similarity of the bacterial community structure was high between the wheat-maize rotation with quicklime and the green manure with quicklime. This suggests that quicklime also had a more significant influence on the bacterial community structure of continuous peanut fields during the cultivation year.

The fungal communities showed different patterns from bacteria. The separated PCoA plot (Figure 5B) showed that, regardless of cropping year, the fungal communities of each treatment were divided into two groups: soil under the green manure rotation and soil under the wheat-maize rotation. Moreover, under the same rotation pattern, the fungal communities were divided into two distinct groups by the presence or absence of quicklime application. These results indicate that the difference in rotation pattern was the primary driver of fungal community change for fungal communities. Quicklime application is an essential inducer of fungal community change under certain cultivation practices.

Based on the OTU classification results, the species and relative abundance of bacteria and fungi in all samples were analyzed at the phylum level (Figure 5C). Soil bacterial species under all treatments covered twenty-three bacterial phyla and six fungal phyla. Among all treatments, *Actinobacteria*, *Proteobacteria*, and *Chloroflexi* were the dominant soil bacterial taxa. However, bacterial phyla composition and relative abundance differed significantly under different cropping years and cultivation managements. The abundance of *Actinobacteria* varied from 28.70 to 45.34%, while the proportion of *Proteobacteria* in each sample ranged from 22.51 to 28.69%. Different cultivation practices contributed differently to the alteration of the bacterial community structure in terms of the proportion of major bacterial phyla under different treatments. For example, the percentage of *Actinobacteria* in the soil library was lower in the quicklime application than in the non-quicklime treatment. Therefore, the percentage of *Actinobacteria* was significantly reduced by quicklime application treatment. For the fungal community taxonomic composition (Figure 5D), *Ascomycota*, *Basidiomycota*, and *Mortierellomycota* were the three most dominant soil fungal taxa in all treatments. Although the species composition of soil fungal communities was similar at the phylum level under different continuous cultivation patterns, there were significant differences in the relative abundance of major soil fungal genera under different cultivation practices at the genus level. For instance, as a critical class of fungal genera in *Ascomycota*, the relative abundance of *Leptosphaerulina* increased significantly as crop years increased. However, there was a decreasing trend under green manure rotation and wheat-maize rotation. Additionally, the relative abundance of *Leptosphaerulina* showed a further decrease in the quicklime-applied soil under the same planting year conditions.

### Correlation analysis between different microbial abundances and soil factors under different cultivation managements

**Figure 6:**
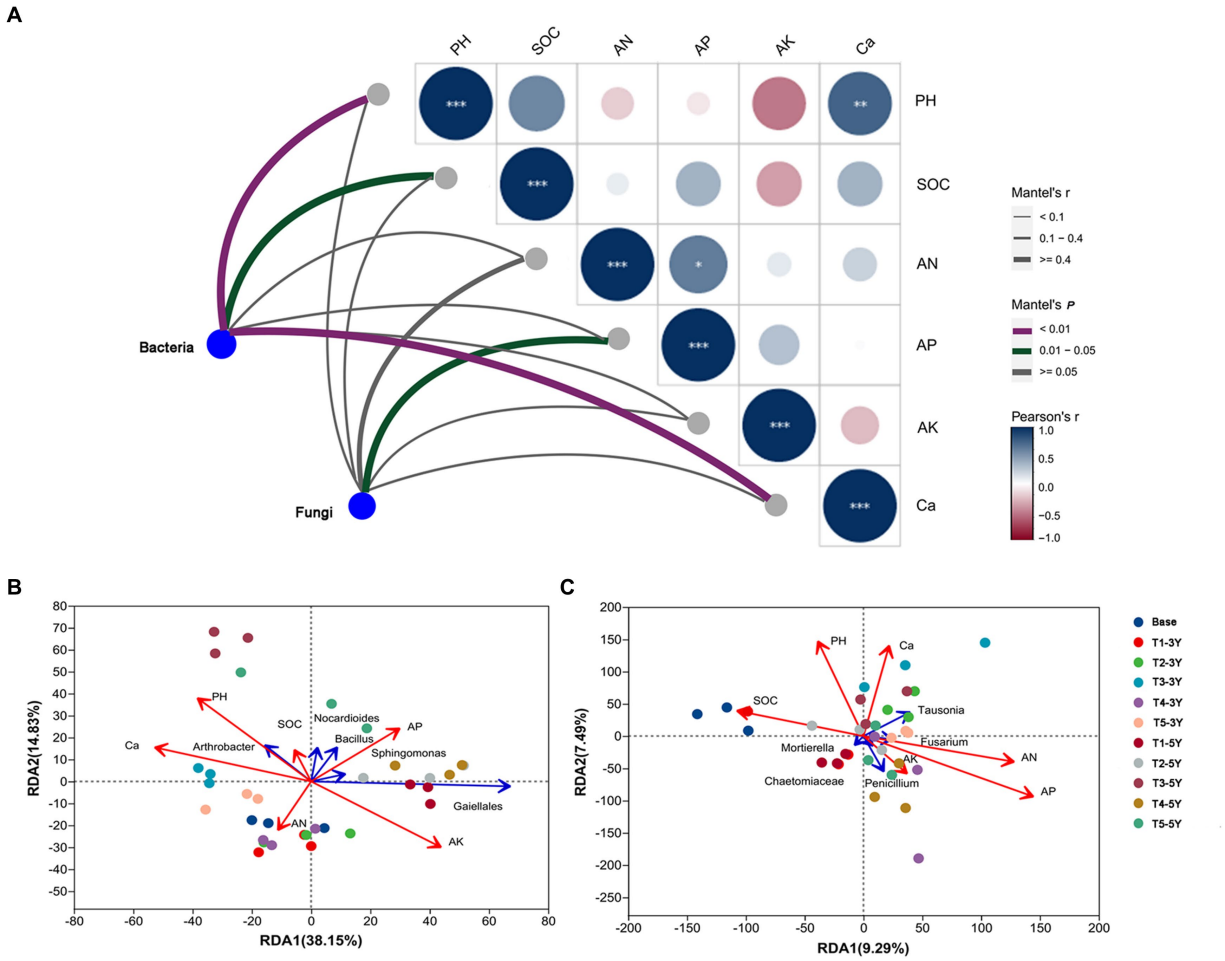
Correlation analysis of bacterial and fungal composition with soil variable factors. **(A)** Mantel tests depicting the association of bacterial composition (16 S OTUs) and fungal composition (ITS OTUs) with soil variable factors. The width of the Mantel edge represents the Mantel *r*-value. The color of the edge represents statistical significance. The color gradient of the Pearson correlation indicates the pairwise correlation of the soil variable factors. The number of asterisks represents the significance level. ^*^0.01 < *p* < 0.05, ^**^0.001 < *p* < 0.01, ^***^p < 0.001. The redundancy analysis (RDA) of bacterial **(B)** and fungal **(C)** communities and soil variable factors. The five most abundant bacterial and fungal genera of bacteria and fungi are shown in this figure.

To measure the relationship between the different microbial communities in the samples and soil environmental variables and to assess the correlation between microbial taxa and soil environmental variables, mantel test analysis was used to clarify which soil chemical factor was the main ecological driver influencing the microbial community composition (Figure 6A). Soil pH (*p* < 0.01), Ca (*p* < 0.01) and SOC (*p* < 0.05) exhibited significant correlations with bacterial community. For fungi, AP in soil (*p* < 0.05) was the main driver of fungal community composition.

We further correlated soil chemical properties with bacterial and fungal communities at the genus level using an RDA test (Figure 6B). Soil chemical properties had a significant effect on the abundance of fungi and bacteria at the genus level. Soil pH and Ca had a significant effect on the distribution of bacterial communities. These results were consistent with the results of the Mantel test, showing that these two soil variables were positively correlated. Soil pH positively correlated with the abundance of *Arthrobacter*, *Bacillus*, and *Nocardioiders*. In contrast, soil pH negatively correlated with the abundance of *Sphingomonas* and *Gaiellales*. Soil Ca content positively correlated with the abundance of *Arthrobacter*, while negatively correlated with the abundance of *Nocardioiders*, *Bacillus*, *Sphingomonas*, and *Gaiellales*. Despite the positive correlation between soil pH and Ca content, the dominant influential factors in the bacterial community, they had different regulatory effects on specific bacterial genera, such as *Bacillus* and *Nocardioiders*. Among the top 50 abundant bacteria, *Arthrobacter*, *Acidobacteriales* and *Elsterales*, showed significant correlations (*p* < 0.05) with both soil Ca and pH, indicating that changes in the abundance of these bacteria were closely related to the application of quicklime (Supplementary Figure S1).

Additionally, the mental test revealed that soil AP was critical variable driving changes in fungal community (Figure 6C). A positive correlation between AP and AN was shown by the RDA results. Both soil AP and AN positively correlated with the abundance of *Penicillium*, *Fasuriam*, *Tausonia*, and *Chaetomiacrae*, but negatively correlated with the abundance of *Mortierella*. Among the top 50 abundant fungi, *Papiliotrema*, *Tremellomycetes*, *Phaeosphaeriopsis*, *Oidiodendron*, and *Chaetomidium* displayed strong correlations (*p* < 0.05) with soil Ca and pH, demonstrating that quicklime treatment had a direct impact on changes in these fungi’ abundance (Supplementary Figure S1).

### Microbial collinearity network structure analysis

**Figure 7:**
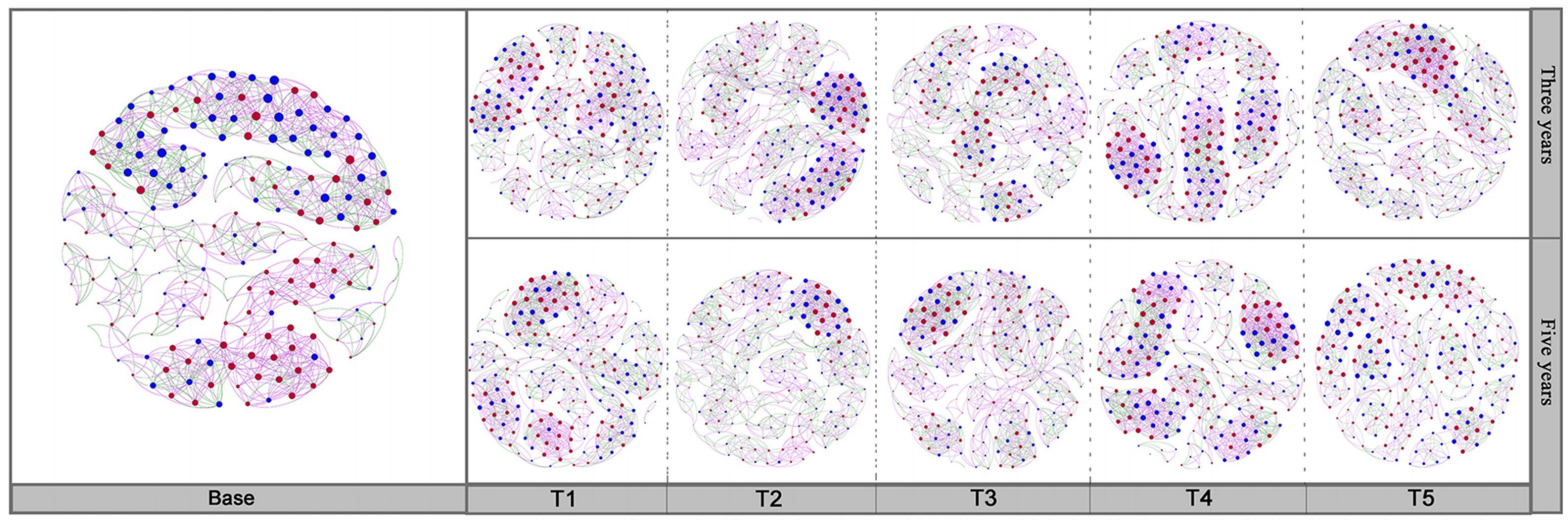
The co-occurrence network of soil bacteria and fungi in each sample. The network was constructed based on a correlation analysis. The nodes represent unique OTUs. The blue node represents bacteria. The red node represents fungi. The size of each node is proportional to its degree. Purple links represent positive correlations. Green links represent negative correlations between nodes.

The interactions between bacterial and fungal communities in soils under different cropping years and cultivation practices were analyzed through co-occurrence networks. The complex relationship between bacteria and fungi was exhibited by calculating the network topology. As shown in Figure 7, the soil bacterial-fungal interactions network in different cultivation practices at the same cropping years were significantly different. The soil bacterial-fungal interactions network depended on different cropping years under the same cultivation managements (Supplementary Table S2). The number of edges had the highest value in the T2-3Y soils compared to other treatments. Microbial networks in soils under different tillage practices were affected by the application of quicklime. Compared to non-quicklime treatments, the proportion of fungal-fungal interactions among all interactions was increased by the application of quicklime. Additionally, the proportion of overall bacterial interactions accounted by bacteria positively associated with bacterial interactions.

The betweenness centrality score could reflect the core microorganisms that play a crucial role in the bacterial-fungal interaction network (Supplementary Table S2). By analyzing the betweenness centrality scores of co-occurrence networks under different treatments, soil bacteria and fungi with the highest abundance differed from these bacteria and fungi with the highest betweenness centrality scores. These results suggested that bacteria and fungi with high abundance did not necessarily play the most critical role in the co-occurrence network. Additionally, the abatement effect of different cultivation managements on the continuous cropping obstacles may depend more on the result of the joint action of multiple microorganisms.

### Effects of different cultivation managements on microbial functional diversity

**Figure 8:**
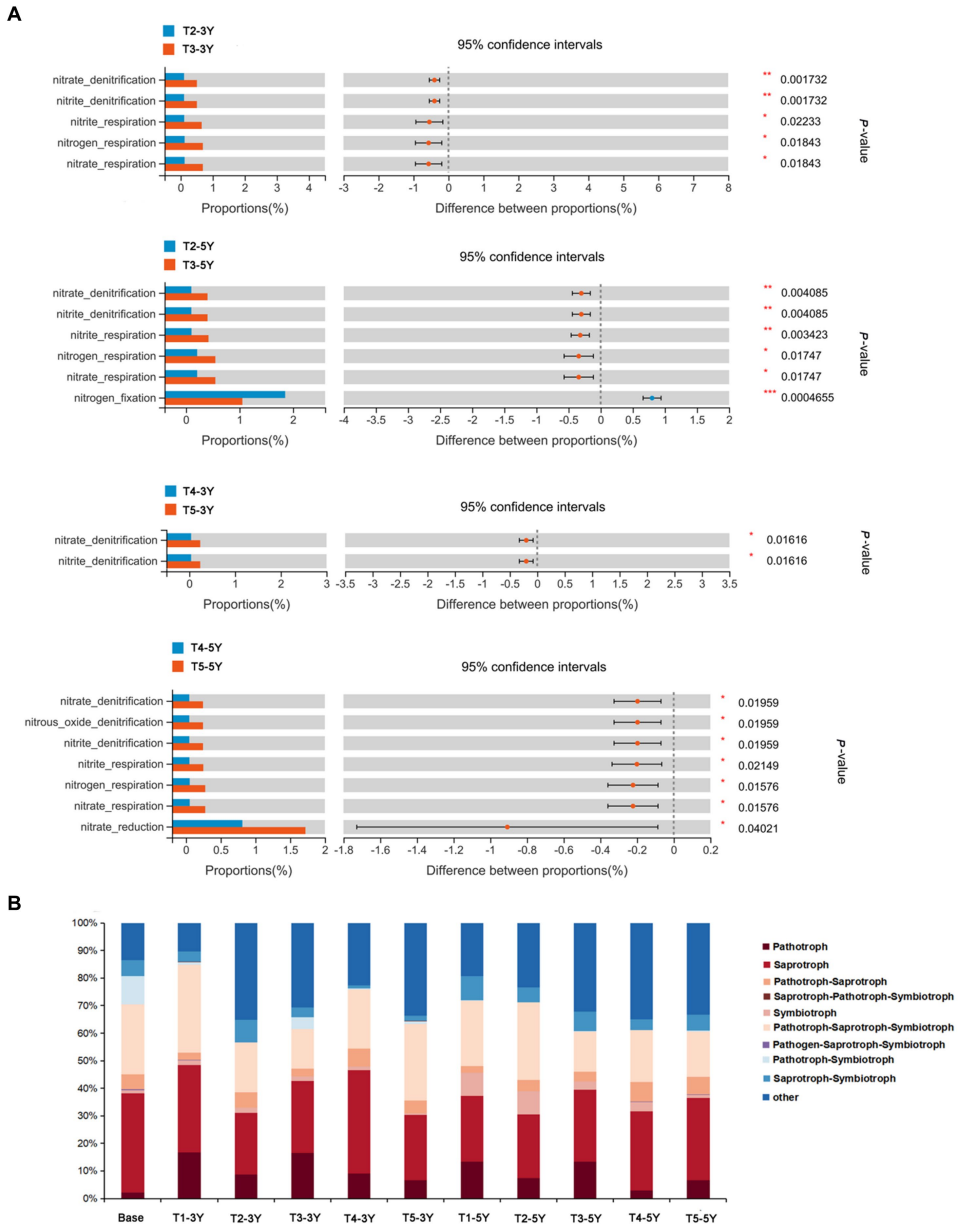
Functions of bacteria and fungi in soils under different cultivation managements at different cropping years. **(A)** Comparison of bacteria associated with nitrogen metabolism in soils in T2-*vs*-T3 and T4-*vs*-T5. Differences between the treatments was measured by Welch’ s *t*-test. The number of asterisks represents the significance level. ^*^0.01 < *p* < 0.05, ^**^0.001 < *p* < 0.01, ^***^p < 0.001. **(B)** Trophic mode of fungi in soil under different treatments. The proportion of different trophic fungi was represented by different colors.

To measure the effect of different cultivation practices used to alleviate continuous cropping obstacles in peanut on the functional diversity of microorganisms, we predicted bacterial and fungal microbial functions in soil by using FAPROTAX database and FUNGuild database, respectively. The FAPROTAX database analysis results indicated significant differences in the metabolic functional groups of soil bacteria under different cultivation practices (Figure 8; Supplementary Table S3). The functions of the bacteria detected in this study were classified into 53 functional descriptions. Among these, green manure rotation and wheat-maize rotation practices increased the functional groups of bacteria with soil chemoheterotrophy (chemoheterotrophy and aerobic_chemoheterotrophy) and nitrogen fixation compared to continuous cropping soils. The addition of quicklime further altered soil functional groups of bacteria under the influence of these two rotation patterns. Adding quicklime significantly changed the functional groups of bacteria related to nitrogen cycling, such as nitrite denitrification, nitrous oxide denitrification, nitrogen fixation, and nitrate respiration, compared to the green manure rotation and wheat-maize rotation practice under non-quicklime application (*p* < 0.05).

In the present study, FUNGuild was used to investigate the functions of the fungi in all soil samples (Figure 8). Results showed that fungal trophic types could be divided into three types: saprotroph, pathotroph, and symbiotroph. The remaining types were complex trophic types. The ecological functional groups of fungal communities differed significantly among different crop years and cultivation practices. As active decomposers in the ecosystem, saprophytic fungi could decompose organic compounds such as plant and animal residues, and play an essential role in soil nutrient cycling. In most samples, saprophytic fungi were the dominant group and showed a decreasing trend with the increase in continuous cropping years. The proportion of saprophytic fungi did not change significantly after green manure measures under 3 years of continuous crop growth. The proportion of saprophytic fungi increased with rotation patterns (green manure rotation and wheat-maize rotation) at the same cropping year. Moreover, the application of quicklime was an essential driver of fungal community change. It was shown by FUNGuild functional predictions that the proportion of the pathotroph-symbiotroph type in fungi was increased by superimposed quicklime compared to a single application of green manure rotation and wheat-maize rotation measures. On the other hand, the proportion of the pathotrophic-saprotrophic fungi decreased compared to a single application of green manure and wheat-maize rotation. These results suggested that quicklime application caused the fungi to adopt a more complex survival strategy and to increase metabolic functional diversity to adapt to changes in the survival environment.

## Discussion

Peanut is one of China’s major oil crops (Yang L. et al., 2022). Due to the extremely limited *per capita* arable land in China, coupled with a single farming system, the proportion of intensive peanut production is increasing (Li et al., 2022). Peanut continuous cropping has led to soil environmental degradation and significant yield reduction. Changes in cultivation managements (including different crop rotation patterns and the addition of soil amendments) have been shown to be effective measures for eliminating continuous cropping obstacles of many crops (Aparicio and Costa, 2007; Zhang et al., 2022a), but the underlying mechanisms remain to be revealed. In the current study, we analyzed the effects of different cultivation managements on peanut yield, soil chemical properties and microbial community dynamics. The results of this study will expand our understanding of the mitigating effects of different cultivation managements on continuous cropping obstacles in peanut, and provide important clues for future research (Wang et al., 2023).

### Wheat-maize-peanut rotation is more beneficial to eliminate continuous cropping obstacles in peanut than green manure-peanut rotation

Crop rotation is an effective agronomic method for improving soil chemical properties and enzyme activity, balancing the ratio of soil nutrients, and increasing crop yield (Venter et al., 2016; Sumner, 2018). Systemic changes in soil ecology brought about by crop rotation, especially its effect on microorganisms, can significantly alleviate continuous cropping obstacles (Shen et al., 2021; Singh and Kumar, 2021; Wang et al., 2022). It has been proven in previous studies that multiple crop rotation can introduce different types of organic residues into the soil, thus increasing the accumulation of organic matter in the soil (Berzsenyi et al., 2000; Govaerts et al., 2008). The process of soil N and P cycling through metabolic compensations is promoted by increasing organic matter, primarily organic C (Vogel et al., 2014). A balanced availability of nutrients is promoted by the coupled cycle of C-N-P (Schmidt et al., 2011), which in turn eliminates continuous cropping obstacles and promotes crop growth. Green manure-peanut rotation and wheat-maize-peanut rotation are two important ways of peanut rotation cropping patterns. As a source of bio-organic fertilizer, green manure has a balanced nutrient content and high fertilizer efficiency, playing an active role in promoting the cultivation of fertility and microbial diversity (Elfstrand et al., 2007; Ye et al., 2014; Du et al., 2020). Ryegrass has well-developed fibrous roots. When used as a green manure measure, its well-developed root system being decomposed in soil can enrich a large amount of nutrients in the tillage layer. This can increase soil organic matter content, enhance soil fertility without applying organic matter, and change the soil microbial community structure (He et al., 2020; Yang et al., 2023). In the current study, available N, P, and K in soil were obviously influenced by green manure-peanut rotation based on RDA results. Compared with continuous cropping, green manure-peanut increased SOC, AN, and AK in soil, and N concentrations in peanut kernels were increased (Figures 2, 3). Additionally, it has been proven in a recent study that the application of ryegrass as green manure could facilitate crop growth by changing bacterial communities. The soil nutrient cycle process was accelerated due to the increased relative abundance of soil microbes associated with nutrition nutrient cycling (e.g., *Proteobacteria*, *Bacteroidetes*, *Acidobacteria*, and *Gemmatimonadetes*) under the application of ryegrass (He et al., 2020). Similarly, it was demonstrated in the FAPROTAX functional analysis used in the current study that bacteria with chemoheterotrophy and nitrogen fixation functions were more pronounced in soil with green manure application (Figure 8; Supplementary Table S3). Moreover, the relative abundance of microorganisms related to nitrogen metabolism (*Actinobacteriota*) and carbon metabolism (*Bacteroidota* and *Myxococcota*) increased with the application of green manure (Figure 5). It was indicated that green manure-peanut rotation increase the available soil nutrients by increasing the relative abundance of soil microorganisms associated with nutrient cycling. Consequently, peanut growth is promoted, alleviating the damage caused by peanut continuous cropping.

It has been established that multi-crop rotations are a more effective cultivation practice to improve the sustainability and stability of agricultural systems than single crop rotation (Liu et al., 2022; Wang et al., 2023). Crop yield benefits could increase with crop species diversity in the crop rotation within a certain range (Smith et al., 2023). In the current study, the average peanut yield increased sequentially under T1 (continuous cropping), T2 (green manure rotation) and T4 (wheat-maize rotation) treatments. SOC, soil AP and AK in wheat-maize rotation soil (T3) were higher than those in continuous cropping soil (T1) and exhibited an increasing trend with increasing cropping years. This result indicated that as continuous cropping obstacle mitigation practice for peanut, the yield enhancement effect of wheat-maize-peanut rotation is stronger than that of green manure-peanut rotation. In the third year of cropping, AP was higher in the T4 treatment than in T2, and in the fifth year of cropping, AN, AP, and AK in the soil were higher in the T4 treatment than in T2. These results indicated that the wheat-maize-peanut rotation is more effective in maintaining and even increasing soil fertility than the green manure-peanut rotation, and that the fertility promotion effect increases with the number of cropping year. This result is most likely due to the introduction of more crops with different nutrient requirements in the T4 crop rotation system, resulting in a more balanced soil nutrient environment and thus easier soil fertility maintenance (Breza et al., 2022; Liang et al., 2023). It has already been shown in previous studies that multi-crop rotation could enhance soil microbial community diversity, optimize soil microbial community structure and function, and promote soil health (Liu et al., 2022). Li et al. (2022) showed that changes in soil microbial community composition (with an increase in beneficial microorganisms and a decrease in pathogenic microorganisms) and soil properties caused by multi-crop rotation could alleviate continuous cropping obstacles of peanut. In our results, the alpha diversity of the bacterial and fungal communities in the T4 soil was higher than that in T1 soil. This is consistent with the finding of a previous study reported by Li et al. (2018), who found that crop rotation could increase the diversity of microorganisms in peanut continuous cropping soil. For bacteria, among the top 10 most abundant phyla, the relative abundance of half of these phyla increased due to T4 treatment. *Actinobacteriota*, *Proteobacteria*, and *Bacteroidota* (Wegner and Liesack, 2016) were reported to have the capability to decompose organic matter and promote humus formation, while *Chloroflexi* and *Myxococcota* are able to promote nitrogen and carbon cycling (Kragelund et al., 2007; Ohore et al., 2022). The increased relative abundance of these microorganisms may become a powerful driving force for soil organic matter and nutrient cycling and transformation, and to accelerate the transformation and supply of soil nutrients, promote the peanut development. The increase in pathogenic fungi was considered one of the critical causes of continuous cropping obstacles. Multi-crop rotation has become an important method for managing soil-borne fungal diseases, in part because of its ability to increase the diversity of soil fungal community. Diverse fungal community could contribute to reducing pathogenic fungi through mechanisms such as competition, antagonism, and induce systemic resistance, thereby reducing crop disease incidence (Govaerts et al., 2008; Bainard et al., 2017; Frąc et al., 2018; Liu et al., 2020). In this study, we found that the diversity of fungi was increased under T4 treatment. This increase in diversity may have improved functional adaptability or resistance to disturbance of the peanut cropping system. Additionally, according to the results of the co-occurrence network analysis, the implementation of T4 increased the number of interactions between fungi, which in turn increased the competition between fungi. Therefore, we believe that wheat-maize rotation can suppress the accumulation of pathogenic fungi by enhancing mutual competition among fungi, thus building a soil environment conducive to peanut growth and development.

### The application of quicklime is an efficient measure to further alleviate continuous cropping obstacles in peanut

Peanut is a crop sensitive to continuous cropping (Chen et al., 2012; Li et al., 2014a). One of the fundamental reasons for the continuous cropping obstacles is the self-toxic effect caused by secretion from the peanut root system. Organic acids, such as phenolic, cetyl, and oleic acids, have been shown in previous studies to be the primary peanut chemosensitive substances in continuous cropping soils (Liu et al., 2010; Li et al., 2014a). Organic acid accumulation occurs with continuous cropping, creating an acidic soil environment. Soil acidification makes peanut more susceptible to pathogenic bacterial and fungal infestations (Zhou et al., 2014). Applying quicklime is effective to increase soil pH (Mayfield et al., 2004; Liang et al., 2021; Zhao et al., 2022). In the present study, we found that quicklime superimposed with T2 and T4 treatments increased peanut yield compared with these two treatments alone. Presumably, this occurs because residues from green manure and wheat-maize rotation have smaller-molecule organic acids during decomposition. The organic acids produced by the decomposition of root residues generated during green manure and crop rotation application could be neutralized by quicklime application. Consequently, the self-toxic effects of organic acids in the soil on peanut could be alleviated, facilitating the development of green manure and wheat-maize rotation potential.

Calcium is one of the essential nutrients in plant growth and development and has the functions of stabilizing cell membranes and cell walls and enhancing plant tolerance against environmental stresses (Lee and Seo, 2021). In addition, Ca^2+^, as an essential second messenger of signal transduction, could participate in several physiological and biochemical processes of plant growth, development, and stress resistance (Yang S. et al., 2022). Peanut is a calcium-sensitive crop. It has been shown in previous studies that when peanut is deficient in calcium, photosynthetic products do not function well, and the photosynthetic material conversion rate is lowered. Peanut pod development was hindered, and the kernel became smaller when there is a lack of Ca^2+^ (Yang et al., 2017). Applying exogenous calcium to peanut can promote the transfer of nutrients from plant roots and stems to pods, regulate peanut growth, and improve peanut yield and quality (Basu et al., 2008). In this study, we found that a large amount of Ca^2+^ was introduced by applying quicklime, resulting in a significant increase in soil calcium content and peanut plant calcium uptake, improved peanut growth, and increased yield.

The plant growth environment is closely related to the microflora in the soil, and its microbial community dynamics are likely to directly affect plant health and efficient nutrient utilization. It has been proven in a recent study that the application of quicklime has a significant effect on changing the relative abundance of beneficial and pathogenic microorganisms in the soil (Zhang et al., 2022b). This can stimulate the potential of soil microorganisms to promote plant growth. Using high-throughput sequencing, Shen et al. (2018) found that quicklime effectively increased the abundance and diversity of inter-root soil bacterial community, and alleviated the continuous cropping obstacles by suppressing pathogenic microbes such as *Aeromonas*, *Pseudoxanthomonas* and *Saccharomyces* under tobacco plantation. Previous studies showed that soil microbial types and community composition could be significantly altered by the exogenous application of calcium, which in turn affects the resistance, growth, and development of peanut (Ci et al., 2020). Haggag and Timmusk (2008) showed that *Paenibacillus polymyxa* was effective in the biocontrol of peanut crown rot disease. *Lysinibacillus* and *Pseudomonas* were proven had the ability to inhibit aflatoxin activity, degrade aflatoxin or produce aflatoxin inhibitors (Wang et al., 2013; Gong et al., 2019). In this study, the Mantel test revealed that variation in bacterial community abundance was significantly correlated with soil Ca^2+^ concentration. Compared with the group without quicklime application, the relative abundance of peanut growth beneficial bacteria in the soil, such as *Paenibacillus*, *Lysinibacillus*, and *Pseudomonas*, increased with quicklime application. The increased relative abundance of these bacterial genera may promote peanut growth by reducing the occurrence of peanut crown rot disease and aflatoxin infection. Fungal diseases are the largest category of plant diseases caused by pathogenic microorganisms. Li et al. (2014b) proved that years of continuous peanut crops could significantly result in the accumulation of pathogenic microorganisms and decrease the abundance of plant-beneficial fungi in the soil, leading to reduced peanut yields. *Penicillium* and *Fusarium*, critical pathogenic fungi, have been reported to cause peanut fruit rot and root rot diseases (Rajeendran et al., 2017). The results of the present study showed that *Penicillium* and *Fusarium* were the dominant genera of the soil fungal community (Figure 5). The relative abundance of *Penicillium* and *Fusarium* was lower in the quicklime application than in the non-quicklime application. The decline of pathogenic fungi in continuous cropping soil reduced soil-borne diseases and might be one of the fundamental reasons for the improved peanut yield under quicklime application. In the present study, the combined application of quicklime with cultivation practice increased peanut yield more than the cultivation practice alone. *Arthrobacter*, *Bryobacter*, *Candidatus*_*Solibacter*, and *Acidothermus* are the key genera for decomposing organic matter and utilizing carbon sources in soil (Rime et al., 2015; Yu et al., 2016). In our results, by analyzing the correlation between microbial community change and cultivation measure, we found that the community changes in *Arthrobacter, Bryobacter, Candidatus*_*Solibacter*, and *Acidothermus* exhibited a significant correlation (*p* ≤ 0.05) with the calcium content of soil. We speculated that quicklime application might enhance the decomposition of organic matter in soil and the utilization of carbon sources by altering soil bacterial community, thus improving the growth of peanut and alleviating continuous cropping obstacles.

In addition, it is worth noting that while crop rotation (T2, T4) and crop rotation in combined with quicklime application (T3, T5) are superior to continuous cropping (T1) in guaranteeing peanut yields, there is a tendency for the yield advantages of these four cultivation managements tend to decline year after year. It has been shown that different crop rotation patterns have different soil mineral nutrient content and soil water retention capacity due to different cultivated crops (Zhang et al., 2019; Cui and Yan, 2022). Although crop rotation plays an important role in improving the multifunctionality of soil ecosystems and maintaining the yield stability of the main crop, for main crop with sensitive mineral fertilizer and water requirements, multi-year crop rotation cannot make up for the differences in mineral fertilizer requirements and water-fertilizer coupling effects of the main crop, and the differences in different crop rotation patterns will decrease with the increase in the number of cropping years (Liu et al., 2023). In addition, the effects of different soil types on the stable yield effect of crop rotation are different (Van Doren Jr et al., 1976; Fiorini et al., 2020). In the future, it is still necessary to further explore the optimization of fertilization and water supply measures under different soil types, combined with different crop rotation systems on the sustainability of stable crop yield. Nevertheless, without considering other factors, the results of the present study suggested that wheat-maize-peanut rotation with quicklime application is an effective peanut yield stabilization measure than the other four cultivation measures.

## Conclusion

This study provides evidence that cultivation management plays a significant role in changing soil chemistry and shaping the composition and functions of bacterial and fungal communities on a time scale. Peanut continuous cropping leads to increased soil acidification, reduced soil fertility and induced an increase in pathogenic bacteria and fungi. Compared with peanut continuous cropping, the application of crop rotation increased microbial diversity and effectively enhanced the microbial functions associated with carbon and nitrogen cycling. Importantly, crop rotation with quicklime application effectively modifies the soil acidification environment, increases contents of Ca and SOC in soil and changes the relative abundance of potentially beneficial and pathogenic bacteria and fungi, thereby promoting plant development and yield of peanut. Moreover, variation in bacterial community is mainly attribute to soil pH, Ca and SOC content, while variation in fungal community was more closely related to soil P content. In addition, compared to peanut continuous cropping, the application of green manure-peanut rotation and wheat-maize-peanut rotation increase peanut yield by 40.59 and 81.95%, respectively. Whereas the combination of these two rotation patterns with quicklime application further increase peanut yield by 28.76 and 24.34%. Peanut yield was highest in wheat-maize-peanut rotation combined with quicklime application treatment. Therefore, during actual production, the combination of wheat-maize-peanut rotation with quicklime application was an effective measure to improve soil fertility and microecology, alleviate peanut cropping obstacles and promote the sustainable development of peanut production. Since this study was conducted only in peanut fields with brown soil, additional investigations in various agricultural regions are still required to verify the effectiveness of this measure.

## Data availability statement

The datasets presented in this study can be found in online repositories. The names of the repository/repositories and accession number(s) can be found below: https://www.ncbi.nlm.nih.gov/, PRJNA901660.

## Author contributions

LY: Writing – original draft, Writing – review & editing. CW: Conceptualization, Investigation, Supervision, Writing – review & editing. XH: Project administration, Validation, Writing – review & editing. HL: Data curation, Formal analysis, Methodology, Software, Writing – review & editing. QW: Data curation, Formal analysis, Methodology, Writing – review & editing. XS: Investigation, Methodology, Writing – review & editing. ML: Validation, Writing – review & editing. PS: Writing – review & editing, Writing – original draft.

## Footnotes

^1^ http://www.arb-silva.de

^2^ http://unite.ut.ee/index.php

^3^ http://www.loucalab.com/archive/FAPROTAX/lib/php/index.php?section=Home

^4^ http://www.stbates.org/guilds/app.php
