# Supplementary figures and images for: Multi-year crop rotation and quicklime application promote stable peanut yield and high nutrient-use efficiency by regulating soil nutrient availability and bacterial/fungal community
Source: Front Microbiol. 2024 May 17;15:1367184. doi: 10.3389/fmicb.2024.1367184 (PMC11140132; doi:10.3389/fmicb.2024.1367184)

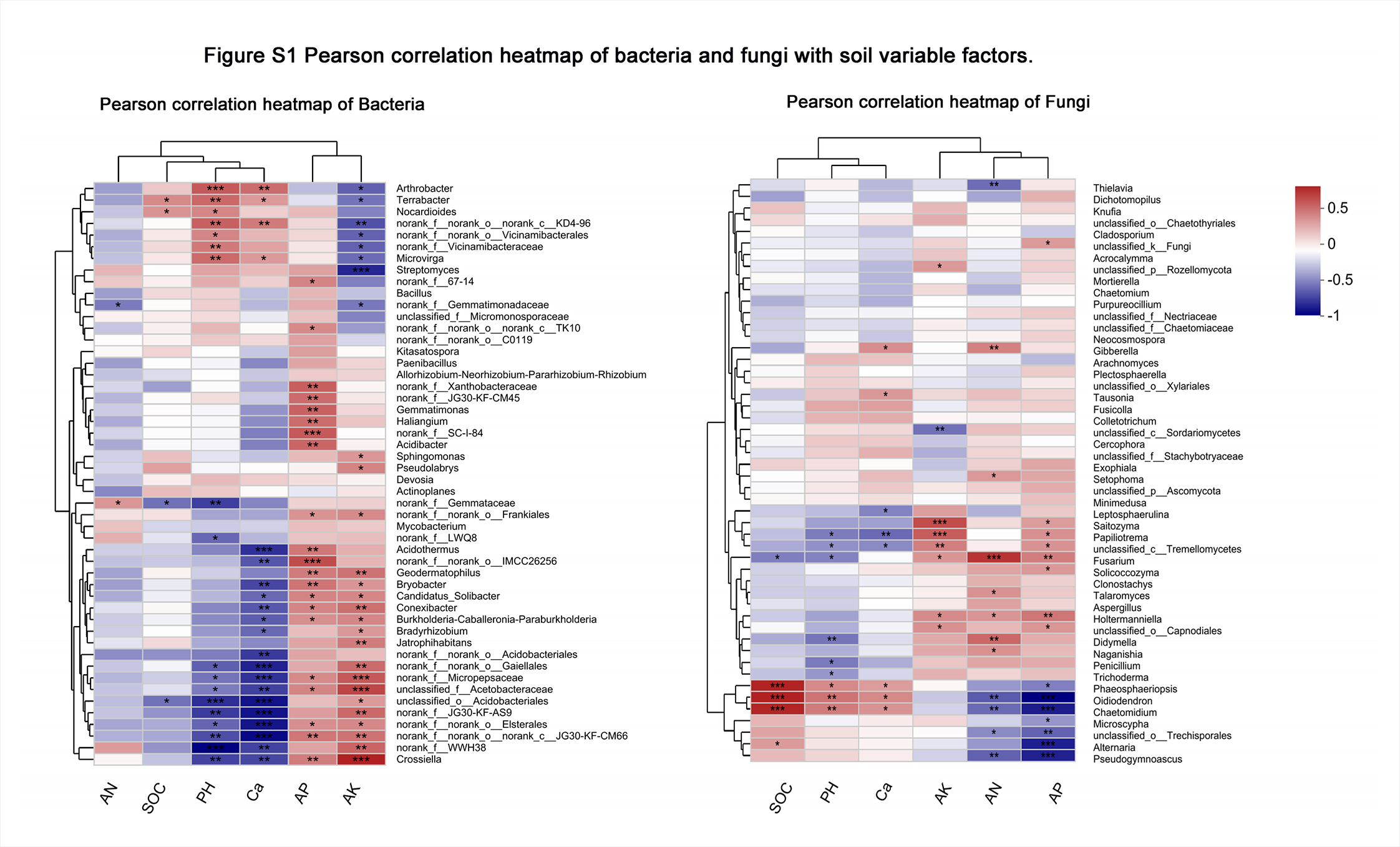

Supplement: Supplementary file 1 [file Image_1.TIF]
